# Revolutionizing outcomes: endoscopic ultrasound-guided gallbladder drainage using innovative electrocautery enhanced-lumen apposing metal stents for high-risk surgical patients

**DOI:** 10.1038/s41598-024-63608-5

**Published:** 2024-06-05

**Authors:** Hyung Ku Chon, Yun Chae Lee, Tae Hyeon Kim, Seung Ok Lee, Seong-Hun Kim

**Affiliations:** 1 Division of Biliopancreas, Department of Internal Medicine, Wonkwang University College of Medicine, Iksan, Republic of Korea; 2 Institute of Wonkwang Medical Science, Iksan, Republic of Korea; 3 Division of Gastroenterology, Department of Internal Medicine, Jeonbuk National University Hospital, Jeonju, Republic of Korea; 4 Research Institute of Clinical Medicine, Biomedical Research Institute of Jeonbuk National University Hospital, Jeonbuk National University, Jeonju, Republic of Korea

**Keywords:** Cholecystitis, Acute, Endosonography, Gallbladder, Drainage, Diseases, Medical research

## Abstract

This study retrospectively evaluated the outcomes of endoscopic ultrasound-guided gallbladder drainage (EUS-GBD) using novel electrocautery-enhanced lumen-apposing metal stents (LAMS) in high-risk patients with acute cholecystitis (AC). Between January 1, 2021, and November 30, 2022, 58 high-risk surgical patients with AC underwent EUS-GBD with the novel electrocautery-enhanced LAMS. The technical success rate was 94.8% (55/58), with one case of duodenal perforation requiring surgery with complete stent migration and two of partial stent migration into the gallbladder. However, the clinical success rate was 100% (55/55). Recurrent AC occurred in 3.6% of the cases (2/55), managed with double pigtail plastic stents through the LAMS. Early AEs observed in 1.8% (1/55) due to stent obstruction. Late AEs occurred in 5.4% (3/55), including two cases of cholangitis and one of stent obstruction. For 33 patients followed over 6 months, LAMS maintenance was sustained in 30 cases. Two patients underwent double-pigtail plastic stent replacement after LAMS removal, and one underwent LAMS removal during surgery following tumor stage regression after chemotherapy for cholangiocarcinoma. The novel electrocautery-enhanced LAMS demonstrated high technical and clinical success rates in high-risk surgical patients with AC, maintaining effective gallbladder drainage with minimal AEs during long-term follow-up, thus highlighting its efficacy and safety in challenging patients.

## Introduction

Acute cholecystitis (AC) poses significant clinical challenges and often requires prompt treatment^1^. Traditional management strategies, such as percutaneous transhepatic gallbladder drainage (PTGBD) and laparoscopic cholecystectomy, may not be suitable for all patients, especially those with high surgical risk or complex anatomical considerations.

Endoscopic gallbladder drainage, including endoscopic transpapillary gallbladder drainage (ETGBD) and endoscopic ultrasound-guided gallbladder drainage (EUS-GBD), has emerged as a promising alternative. Moreover, the procedure has demonstrated efficacy in various clinical scenarios^2–6^. ETGBD involves access to the gallbladder through the cystic and bile ducts, whereas EUS-GBD uses EUS guidance to create a direct transluminal route for gallbladder drainage. Both techniques have demonstrated promise in achieving successful gallbladder decompression and effectively alleviating symptoms. However, a recent systematic review indicated that compared to ETGBD, EUS-GBD has higher technical and clinical success rates and a lower recurrence rate^7^.

Advancements in the EUS-GBD procedure have occurred since the initial use of a 7Fr double pigtail, including the development of modified covered self-expandable metallic stents or lumen-apposing metal stents (LAMS)^8–10^. The dumbbell-shaped LAMS adheres to the intestinal or gastric walls, as well as the gallbladder wall, thereby reducing the risk of stent dislodgement or bile leakage. Furthermore, the development of an electrocautery-enhanced LAMS, equipped with an electrical cauterization function at the tip of the LAMS delivery system, allows for single-step insertion of the LAMS without the need for an additional dilating device for the fistulous tract, rendering the procedure convenient^11^.

**Figure 1:**
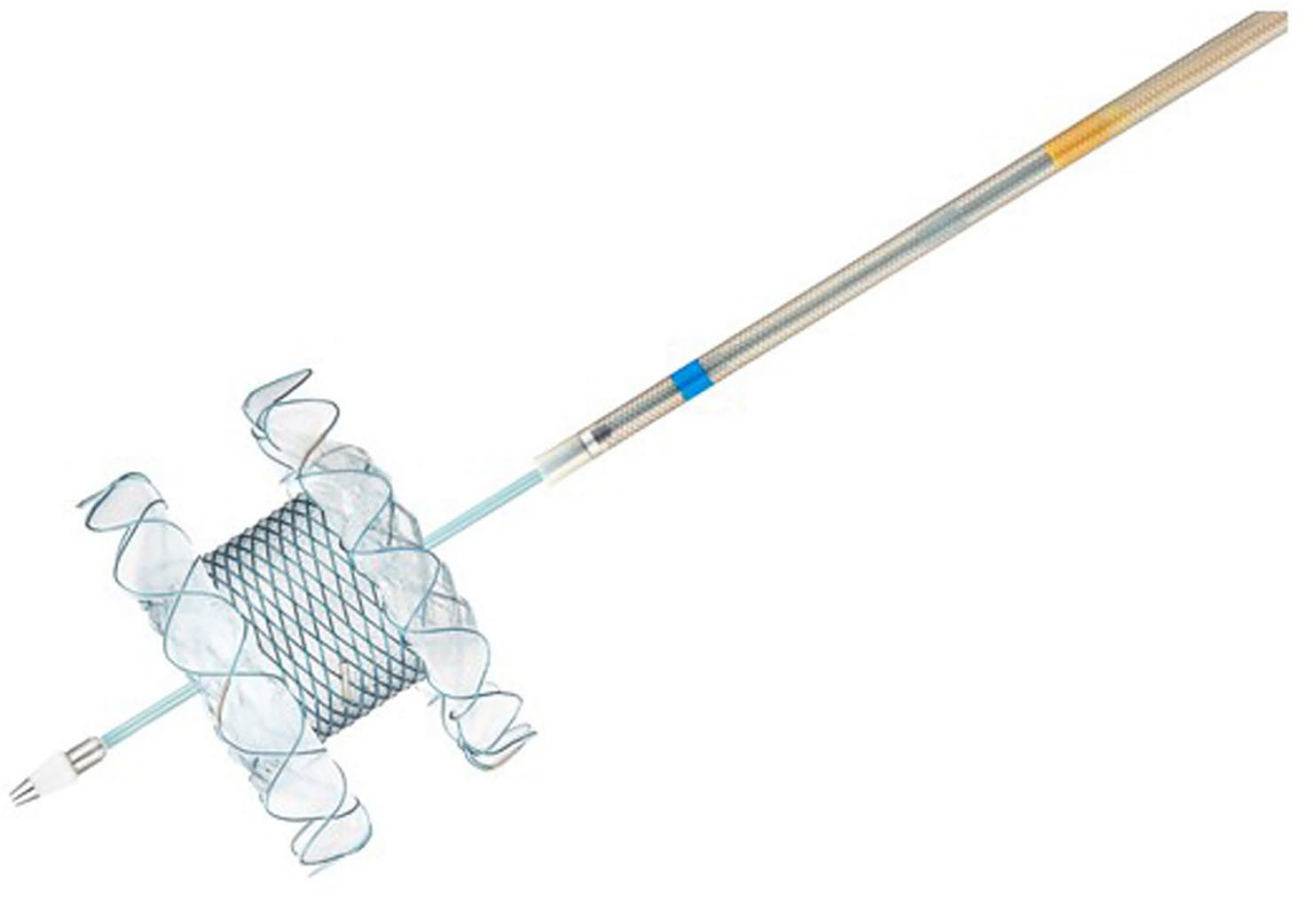
Novel lumen apposing metal stents with a flexible flare design and an electrocautery-enabled delivery system (Hot-SPAXUS; Taewoong Medical Co., Goyang-si, Korea).

Recently, the newly developed Hot-SPAXUS (Taewoong Medical Co., Goyang-si, Korea), featuring a flexible flare design and an electrocautery-enabled delivery system, has been used in various EUS-guided interventions (Fig. 1). However, research on EUS-GBD using Hot SPAXUS is limited.

This study aimed to evaluate the procedural outcomes, long-term efficacy, and safety of EUS-GBD using Hot-SPAXUS in high-risk surgical patients with AC.

## Methods

### Patients

All consecutive high-risk surgical patients with AC, based on the Tokyo Guidelines 2018^12^, who underwent EUS-GBD with Hot-SPAXUS at a tertiary center were retrospectively analyzed between January 1, 2021, and November 30, 2022. Follow-up assessments were conducted until November 30, 2023, or death. The inclusion criteria were as follows: (1) AC, (2) high-risk surgery, (3) unsuccessful and/or failed ETGBD, (4) lack of consent for PTGBD, and (5) written informed consent. The exclusion criteria were as follows: (1) age < 20 years, (2) lack of consent for EUS-GBD with Hot-SPAXUS, (3) pregnancy, and (4) loss to follow-up until September 30, 2023. The assessment of patients' surgical risk involved a multidisciplinary team comprising an anesthesiologist, a surgeon, and a physician. High risk for surgery was considered based on the presence of one or more of the following criteria: age ≥ 80 years, American Society of Anesthesiologists (ASA) grade 3 or higher, or age-adjusted Charlson Co-morbidity Index (CCI) score ≥ 4. We contacted patients or their relatives via phone to verify the progression in cases not accessible through electronic medical records. The current study was approved by Institutional Review Board (IRB No. CUH 2023–12-021) and was conducted in accordance with the principles of the Declaration of Helsinki (revised in Edinburgh 2000).

### The gallbladder drainage procedure

After informed consent was obtained, antibiotics were administered to all patients before the procedure. The procedures were conducted by experienced endosonographer (SH.K.) with expertise in both ERCP and EUS, each performing more than 150 EUS procedures annually for pancreaticobiliary diseases. Patients were placed in the left lateral decubitus or prone position under conscious sedation using intravenous midazolam, meperidine, or occasionally propofol.

**Figure 2:**
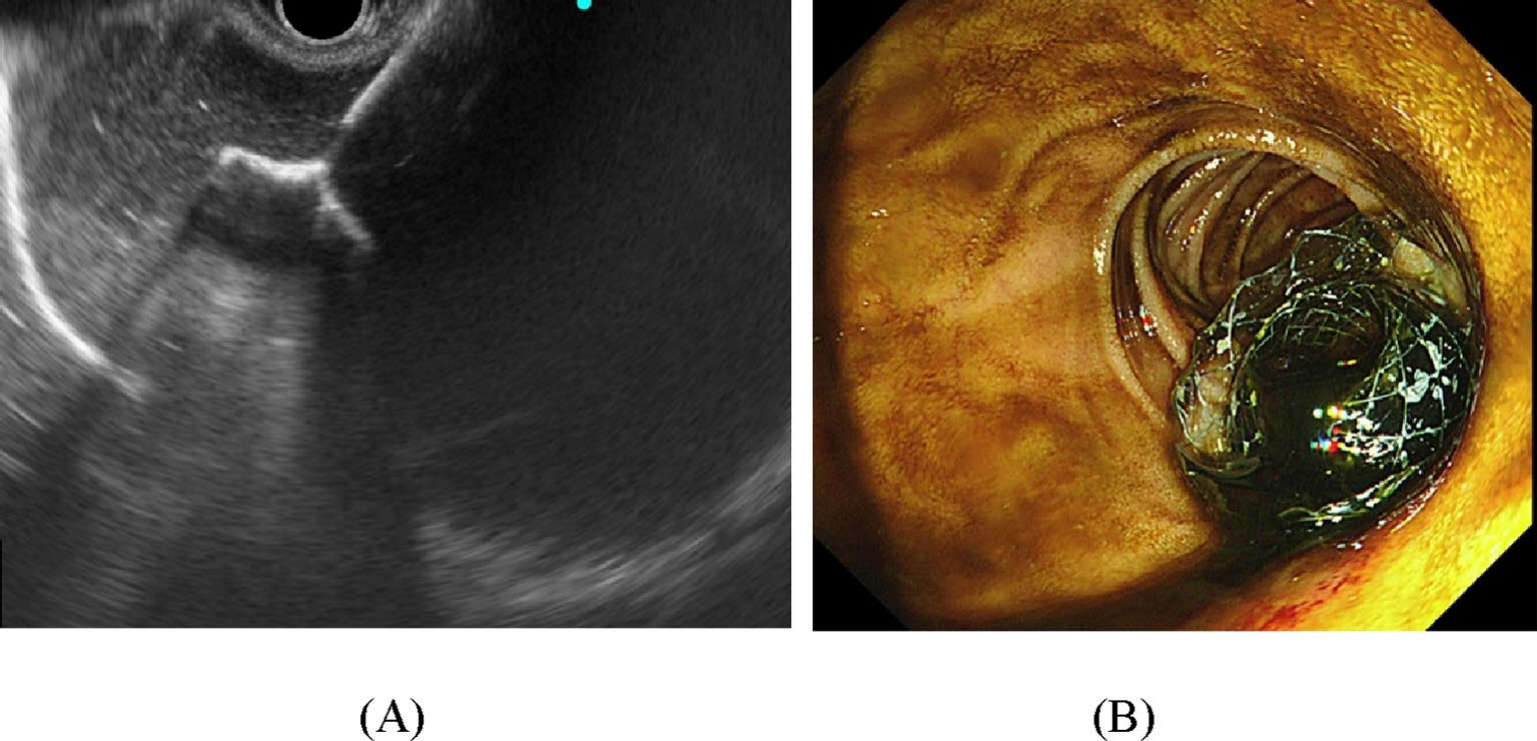
(**A**) Endoscopic ultrasound image displaying the distal flange of a lumen-apposing metal stent (LAMS) deployed into a distended gallbladder with sludge. (**B**) Endoscopic view of the proximal flange of the LAMS deployed into the gallbladder draining turbid bile fluid.

Therapeutic linear EUS (GF-UCT 260; Olympus Medical Systems, Tokyo, Japan) was used in all procedures and was advanced into the prepyloric gastric antrum or duodenal bulb to obtain the optimal puncture site for the gallbladder neck or body, avoiding any intervening blood vessels. The freehand technique (Video 1) or two-step technique (Video 2), which involved puncturing with a 19-gauge needle (EZ shot3 plus; Olympus Medical Systems, Tokyo, Japan) followed by the placement of a guidewire (0.025-inch VisiGlide; Olympus Medical Systems), was performed for the placement of Hot-SPAXUS. The proximal flange of the stent was deployed and the released stent was guided under EUS to adhere to the gallbladder wall. Subsequently, the distal flange was released using the "intrachannel technique" which was then pushed outside by pulling back the echoendoscope under endoscopic view. (Fig. 2) Depending on the operator’s judgment, either a nasogalbladder drainage tube or a double-pigtail plastic stent was inserted through the LAMS, and the procedure was concluded. Subsequently, a peroral cholecystoscopy was not performed.

### Study definition and outcome measures

Technical success was defined as the successful placement of the Hot-SPAXUS between the gallbladder and gastric/duodenal lumen, confirmed by the drainage of bile or purulent discharge. Clinical success was defined as the resolution or significant improvement of clinical symptoms associated with AC, including a reduction in pain and fever, and improvement in inflammatory markers, such as white blood cell count and C-reactive protein, within 7 days of the procedure. Recurrent AC refers to the reappearance or worsening of symptoms, signs, and radiological features compatible with AC after the initial period of improvement or resolution following the procedure. Intraprocedural adverse events (AEs) are complications that occur during the procedure. Early AEs referred to any unexpected or undesirable occurrence within 14 days after the procedure, whereas late AEs included those that occurred 14 days post-procedure. Moreover, AEs were graded according to the endoscopic AEs classification^13^. Long-term was defined as tracking observation for more than 6 months after the success of the procedure.

The primary outcomes evaluated were technical and clinical success rates of EUS-GBD using Hot-SPAXUS. The secondary outcomes included the incidence of recurrent AC and any AEs following the procedure.

## Statistical analyses

Descriptive statistics were used to summarize baseline characteristics of the study population, procedural details, and outcomes. Continuous variables were expressed as median with interquartile range (IQR) or mean with standard deviation (SD) and range, and categorical variables were presented as frequencies and percentages. For comparisons between the techniques, the Mann–Whitney U test was used for continuous variables, and Fisher's exact test or chi-squared test was used for categorical variables, as appropriate. Statistical significance was set at *p* < 0.05. 3. Kaplan–Meier survival analysis was performed to estimate stent patency. All statistical analyses were conducted using SPSS Statistics software (version 22.0; IBM Corp., Armonk, NY, USA).

## Results

### Study population

**Figure 3:**
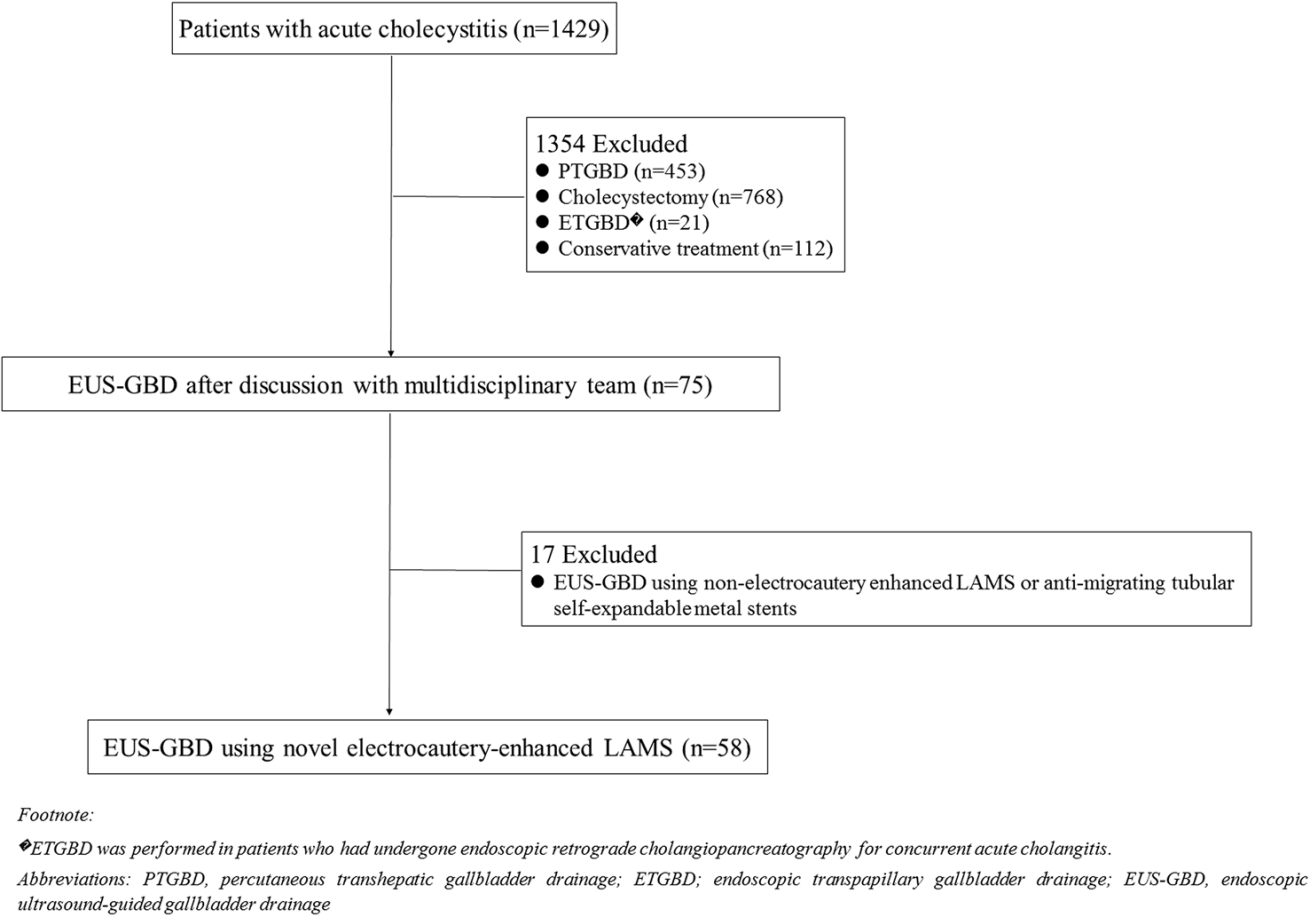
Flowchart of enrolled patients.

During the study period, 58 patients with AC who were at high risk for surgery underwent EUS-GBD using Hot-SPAXUS. (Fig. 3) The baseline characteristics of the 58 patients are presented in Table 1. The median age ± SD of enrolled patients was 81.5 ± 8.6, with 29 males (50.9%). The predominant condition was calculus cholecystitis. Additionally, ASA classes III and IV were observed in 54 and three patients, respectively, and the median age-adjusted CCI ± SD was 8.6 ± 2.5. Patients with underlying malignancies comprised 32 patients (22 with cholangiocarcinoma, two with gallbladder cancer, three with pancreatic cancer, one with ampullary cancer, one with hepatocellular carcinoma, two with sigmoid colon cancer, and one with gastric cancer). Twelve patients with recurrent AC underwent EUS-GBD using Hot-SPAXUS. Among them, ten had previously undergone PTGBD and two had been treated with ETGBD. Biliary metallic stents had been inserted in 17 patients prior to the procedure. The median follow-up period and length of hospital stay after the procedure were 300 days (IQR, 76–464 days) and 9 days (IQR, 7–14 days), respectively.

**Table 1:** Characteristics of 58 patients who underwent endoscopic ultrasound-guided gallbladder drainage with Hot-SPAXUS for acute cholecystitis.

Mean age, years ± SD (range)	81.5 ± 8.6 (54–93)
Male gender, *n* (%)	29 (50.0)
ASA class III/IV, *n* (%)	54/4 (93.1/6.9)
Age-adjusted Charlson comorbidity index, Mean ± SD	8.6 ± 2.5
Antiplatelet or anticoagulant, *n* (%)	19 (32.7)
Underlying malignancy, *n* (%)	
Cholangiocarcinoma	22 (37.9)
Gallbladder cancer	2 (3.4)
Pancreatic cancer	3 (5.2)
Ampullary cancer	1 (1.7)
HCC	1 (1.7)
Others	3 (5.2)
History of acute cholecystitis	12 (20.6)
Acute cholecystitis severity (II/III)	37/21 (63.8/36.2)
Prior biliary metal stent placement	17 (29.3)
Indication of the procedure	
Calculus cholecystitis	38 (65.5)
Acalculus cholecystitis	20 (34.5)
Follow-up, median (IQR), days	300 (76–464)
Hospital stay after procedure, median (IQR), days	9 (7–14)

Other included two cases of metastatic sigmoid colon cancer and one case of metastatic gastric cancer.

SD, standard deviation; HCC: hepatocellular carcinoma; IQR. interquartile range.

### Procedural details

In the majority of the 58 patients, the puncture was performed at the duodenal bulb (96.6%, 56/58) (Table 2). The freehand technique was performed in 23 patients and the two-step technique was used in 35 patients. The predominant size of the Hot-SPAXUS was 20 mm × 8 mm. An additional double-pigtail plastic stents was inserted in one case. The median time from gallbladder puncture to stent deployment was 7 min, while the median time for the freehand technique was 2.5 min, which was significantly shorter than that of the two-step technique (2.5 min vs. 9 min, *p* = *0.018*). No significant difference was observed in the technical success rate between the two techniques (freehand technique: 100% vs. two-step technique: 91.4%,* p* = 0.27).

**Table 2:** Characteristics of the endoscopic ultrasound-guided gallbladder drainage procedure with Hot-SPAXUS for acute cholecystitis.

Puncture site, *n* (%)	
Stomach	2 (3.4)
Duodenum	56 (96.6)
Technique, *n* (%)	
Two-step technique (19G needle, guidewire, then Hot-SPAXUS)	35 (60.3)
Freehand technique (Directly Hot-SPAXUS)	23 (39.7)
Hot-SPAXUS size (length x diameter), *n* (%)	
20 mm × 8 mm	47 (81.0)
20 mm × 10 mm	11 (19.0)
Additional double pigtail plastic stents placed, *n* (%)	1 (1.7)
Additional nasogallbladder drainage tube placed, *n* (%)	17 (29.3)
Time to stent deployment from gallbladder puncture, median (IQR), minutes	7 (3–10)
Two-step technique, median (IQR), minutes	9 (8–11)
Freehand technique, median (IQR), minutes	2.5 (2–3)

The median time to stent deployment from the gallbladder in the freehand technique was significantly shorter than that in the two-step technique (*p* = 018).

IQR. interquartile range.

### Procedural outcomes

**Figure 4:**
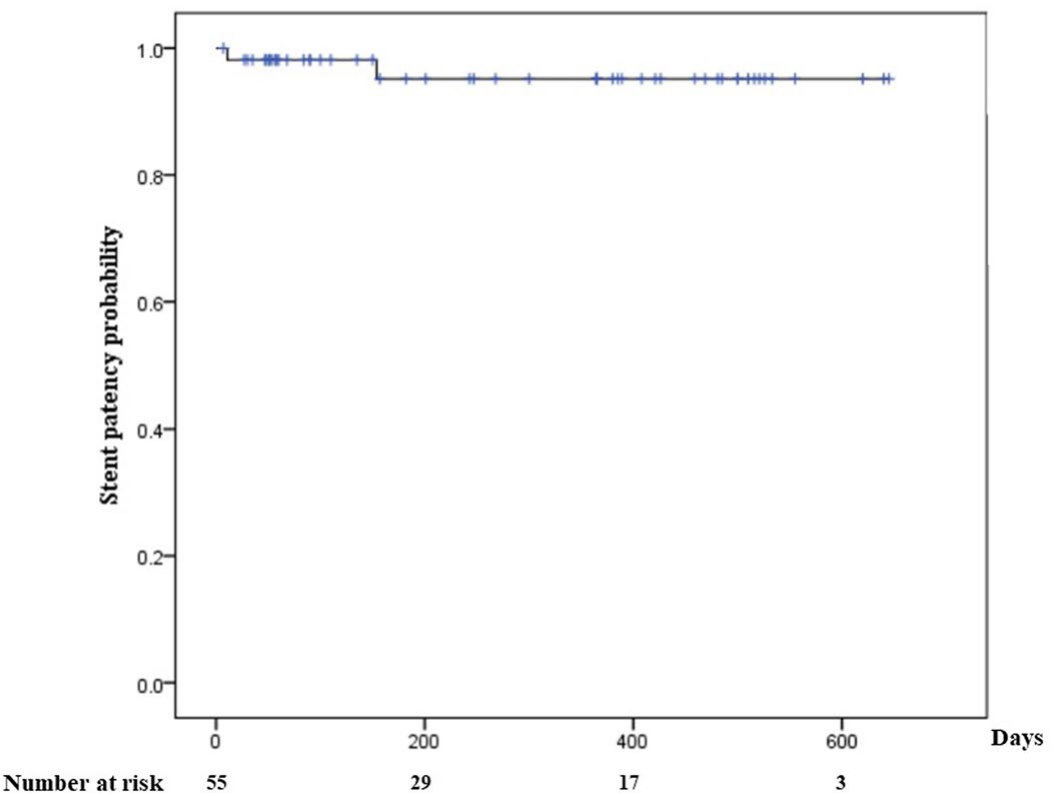
Kaplan–Meier curves for cumulative stent patency of endoscopic ultrasound-guided gallbladder drainage with Hot-SPAXUS.

The technical success rate of the procedure was 94.8% (55/58) as displayed in Table 3. Among the three cases where the procedure failed due to intraprocedural AEs, one involved duodenal perforation with complete internal stent migration into the gallbladder requiring surgery, and the other two had partial internal stent migration into the gallbladder. In two cases with partial stent internal migration, a covered metal stent was inserted along the previously placed guidewire. In the 55 cases with procedural success, the clinical success rate was 100% (55/55). One patient experienced stent obstruction due to food, leading to recurrent AC 11 days after the procedure as an early AE. In the aforementioned patient, balloon sweeping was performed for the obstructed LAMS, and a double-pigtail plastic stents was inserted. Late AEs occurred in three patients. Two developed acute cholangitis and underwent endoscopic retrograde cholangiopancreatography, while one experienced recurrent AC due to stent obstruction. Therefore, a double-pigtail plastic stents was inserted after balloon sweeping with the obstructed LAMS. All AEs were grade III according to the endoscopic AEs classification. Procedural mortality was 0; however, three patients experienced 30-day mortality, one due to pneumonia and two due to the progression of cholangiocarcinoma. The 6-month mortality was observed in 22 patients, all due to the deterioration of the underlying disease or pneumonia unrelated to AC (progression of underlying malignancy, 16; pneumonia, five; cerebral infarction one). Figure 4 displays the cumulative stent patency curve estimated by Kaplan–Meier analysis.

**Table 3:** Outcomes of the endoscopic ultrasound-guided gallbladder drainage procedure with Hot-SPAXUS for acute cholecystitis.

Technical success	55/58 (94.8)
Clinical success (per protocol)	57/58 (98.2)
Clinical success (intention to treat)	55/55 (100)
Recurrent acute cholecystitis, %	2/55 (3.6)
Re-intervention, %	2/55 (3.6)
Intraprocedural adverse events	3/58 (5.2)
Perforation	1 (1.7)
Stent migration	2 (3.4)
Early adverse events	
Stent obstruction	1/55 (1.8)
Late adverse events	
Stent obstruction	1/55 (1.8)
Acute cholangitis	2/55 (3.6)
Procedural mortality	0 (0)
30-day mortality^‡^	3/55 (5.5)
6-month mortality^§^	22/55 (40.0)

Data are presented as numbers (%).

All adverse events were grade III, according to the endoscopic adverse event classification.

^‡^The 30-day mortality resulted from one case of pneumonia and two cases from the progression of cholangiocarcinoma.

^§^ The 6-month mortality resulted from deterioration of underlying conditions unrelated to acute cholecystitis (progression of underlying malignancy, 16; pneumonia, five; cerebral infarction, one).

### Long-term follow-up

Thirty-three patients (mean age ± SD 81.5 ± 8.8, males 17) who underwent successful EUS-GBD using Hot-SPAXUS were followed up for more than 6 months (Table 4). The median follow-up period was 426 days (IQR 365–510 days), during which eight patients died due to underlying disease progression or pneumonia (progression of underlying malignancy, 6; pneumonia, two). The LAMS was maintained in 30 patients until the last follow-up. In two cases, double-pigtail plastic stents replacement was performed after LAMS removal, and in one case, the LAMS was removed during surgery following tumor stage regression after chemotherapy in a patient with cholangiocarcinoma. No LAMS migration was observed. The median number of emergency room visits and hospital admissions after the procedure were 1 (IQR, 0–3) and 1 (IQR, 1–3.5), respectively.

**Table 4:** Long-term outcomes after ultrasound-guided gallbladder drainage with Hot-SPAXUS for acute cholecystitis in 33 patients.

Mean age, years ± SD (range)	81.5 ± 8.8 (54–92)
Male gender, *n* (%)	17 (51.5)
Follow-up, median, IQR (range), days	426 (365,510) (182–555)
Maintaining LAMS, *n* (%)	30 (90.9)
Double pigtail plastic stents replacement after LAMS removal, *n* (%)	2 (6.1)
LAMS removal	1^‡^ (3.0)
LAMS migration	0 (0)
Median number of ER visits (IQR)	1 (0,3)
Median number of hospital admission after procedure (IQR)	1 (1,3.5)
Patient status on follow-up, *n* (%)	
Dead^§^	8 (24.2)
Alive	25 (75.8)

Long-term was defined as tracking observation for more than 6 months after the success of the procedure.

^‡^ Removal of LAMS during surgery following tumor stage regression after chemotherapy in a patient with cholangiocarcinoma.

^§^ Eight deaths unrelated to acute cholecystitis were attributed to underlying medical conditions (progression of underlying malignancy, six; pneumonia, two).

SD, standard deviation; IQR. interquartile range; LAMS, lumen-apposing metal stents; ER, emergency room.

## Discussion

In this study, we described the efficacy and safety of EUS-GBD using Hot-SPAXUS in high-risk surgical patients with AC. The baseline characteristics of the enrolled patients highlighted advanced age (median age 81.5 ± 8.6) and a male predominance (50.9%), consistent with the demographic challenges often encountered in this patient group^14,15^. A significant portion of the study population presented with underlying malignancies (55.2%), emphasizing the challenges posed by concurrent conditions in managing AC in high-risk surgical patients. The history of previous treatments with alternative drainage methods, such as PTGBD or ETGBD, in 20.7% of patients highlights the refractory nature of their condition, prompting the exploration of EUS-GBD as a viable alternative.

The primary outcomes of the current study, including technical and clinical success rates, compared favorably with those in the existing literature on EUS-GBD^16^. The technical success rate aligns with or surpasses the reported rates using alternative devices, reflecting the efficacy of Hot-SPAXUS^3,17^. The high success rate is likely attributed not only to the simplified procedure that eliminates the need for fistulous tract dilatation but also to the expertise of the endosonographer. In three cases of intraprocedural AEs leading to technical failure, stent migration into the gallbladder occurred during stent deployment. This phenomenon was limited to the first five procedures and ceased as the operators became familiar with the stent delivery system.

Factors contributing to stent migration during the procedure, including inadequate stent fixation, technical errors, anatomical variations, and patient-related factors, may be considered. In current study, among the three cases where the procedure failed due to intraprocedural AEs, one involved duodenal perforation with complete stent migration into the gallbladder requiring surgery, and the other two had partial stent migration into the gallbladder. In a case of duodenal perforation, the patient suddenly twisted their body during stent deployment, causing instability of the endoscope, resulting in the entire stent entering the gallbladder. Consequently, the guidewire also slipped out, making salvage procedures difficult, ultimately necessitating surgery. In the other two cases, despite using the “intrachannel technique” to place the stent, inadequate coordination with the assistant during the final stent release resulted in the proximal flange not fully protruding into the intestinal side. This required the insertion of an additional covered metal stent. Based on these experiences, for EUS-GBD with Hot-SPAXUS procedures, adequate sedation is necessary, and proper coordination with the assistant is essential. Even during the "intrachannel technique", endoscopic visualization should confirm the appropriate positioning of the proximal flange of the LAMS.

Among the 55 cases with procedural success, the clinical success rate reached 100%, emphasizing the positive outcomes of the intervention in resolving or significantly improving clinical symptoms associated with AC. In comparison to other studies, the results of this study demonstrated a higher clinical success rate^16,18,19^. This may be attributed to factors such as the post-procedural insertion of a nasogallbladder drainage tube with saline irrigation, and various definitions of clinical success in different studies, influencing the outcomes.

Stent obstruction due to food material, leading to recurrent AC, was observed in two patients at 11 and 154 days post-procedure. The obstruction was addressed through balloon sweeping of the obstructed LAMS, followed by the insertion of a double-pigtail plastic stents. These patients, aged 92 and 91 years, remained recurrence-free during outpatient follow-up after the procedure. Based on these findings, we suggest the placement of a double-pigtail plastic stents through the LAMS for prolonged maintenance. This strategy has the theoretical advantage of preventing bleeding caused by repetitive friction of the inner edge of the LAMS against the gallbladder mucosa^20^.

Procedural mortality remained zero, indicating the absence of immediate fatalities related to the interventions. However, three patients experienced 30-day mortality, and 6-month mortality was observed in 22 cases. Notably, the causes of mortality were associated with the deterioration of underlying diseases or pneumonia unrelated to AC, emphasizing the impact of underlying conditions rather than the procedure itself on patient outcomes.

These procedural details highlight the technical aspects of EUS-GBD using Hot-SPAXUS. Most punctures were performed in the duodenal bulb, demonstrating a consistent approach in this patient cohort^20^. The utilization of both freehand and two-step techniques reflected procedural flexibility and proved effective in achieving successful gallbladder drainage using Hot-SPAXUS in high-risk surgical patients with AC. As the operator's proficiency with the device and procedure improved, the freehand technique became suitable for straightforward cases where access to the target site was relatively easy. Conversely, the two-step technique remained preferred for cases requiring precise needle placement and controlled guidewire advancement, particularly in patients with difficult access. In our study, the initial 20 procedures exclusively utilized a two-step technique.

The freehand technique demonstrated comparable technical success rates and short median procedure times (2.5 min vs. 9 min, *p* = *0.018*), indicating potential procedural efficiency advantages. However, further investigation is warranted to delineate the specific merits of each technique. Considering the experience of partial salvaging of LAMS migration into the gallbladder by inserting a covered metal stent through the guidewire in the two-step technique, the two-step technique may be preferable when the endosonographer is unfamiliar with Hot-SPAXUS. With accumulation of experience in EUS-GBD using Hot-SPAXUS, the freehand technique is being increasingly favored over the two-step technique in recent practice. In cases where endoscope instability is anticipated, the procedure involved inserting the guidewire through the stent delivery system into the gallbladder after directly puncturing both the intestinal lumen and the gallbladder using Hot-SPAXUS. This approach was undertaken to address potential intraprocedure AEs, as an alternative to the two-step technique.

In this study, the extended follow-up of 33 patients who underwent successful EUS-GBD using Hot-SPAXUS revealed sustained efficacy over a median period of 426 days (IQR, 365–510 days). Mortality primarily stems from underlying disease progression, underscoring the importance of considering overall health in procedural evaluations. The consistent maintenance of the LAMS until the last follow-up in 30 patients (90.9%) suggests the device's stability and effectiveness in providing a lasting solution for gallbladder drainage. The absence of LAMS migration supported the reliability of the device in preventing stent dislodgement. Interventions such as double-pigtail plastic stents replacement and LAMS removal during surgery demonstrate flexibility in patient management. Moreover, the median number of emergency room visits and hospital admissions after the procedure, ranging from 1 to 3.5, emphasizes the need for continued monitoring and potential interventions. The cumulative stent patency curve estimated by Kaplan–Meier analysis suggests sustained efficacy in maintaining gallbladder drainage over an extended period.

The initial LAMS with an integrated electrocautery was Hot-Axios (Boston Scientific, Marlborough, Mass, USA). Lisotti et al. reported on thirty-four patients with acute cholecystitis who underwent EUS-GBD with Hot-Axios^14^. The technical and clinical success rates were 92% and 88%, respectively, with AEs occurring in 16% of cases. These AEs included bleeding, misdeployment of the distal flange, and stent obstruction. In an international multicenter study involving seventy-five patients undergoing EUS-GBD with Hot-Axios, technical and clinical success rates were 98.7% and 95.9%, respectively^19^. Procedure-related AEs occurred in two patients: one requiring surgery for perforation and another experiencing major bleeding, which was managed conservatively. Recurrent AC was observed in three patients, with stent migration occurring in two cases and one instance of Bouveret syndrome. To date, there hasn’t been any research directly comparing Hot-Axios and Hot-SPAXUS in the context of EUS-GBD. However, our analysis demonstrated that Hot-SPAXUS exhibits similar technical and clinical success rates as Hot-Axios and comparable rates of AEs in patients with AC. Furthermore, long-term outcomes were equivalent to the studies involving Hot-Axios.

Unlike Hot-Axios, Hot-SPAXUS is composed of a nitinol metal mesh and silicone covering. Moreover. Hot-SPAXUS features large flanges at both ends for stent fixation with a rounded margin designed to minimize trauma to the luminal wall. The distance between the flanges of the stent was significant (20 mm), facilitating a convenient approach to the farthest target. During the stent deployment process, the flanges are designed to contract by up to 7 mm as they moisten, ensuring close adherence to both walls and the 10 Fr traditional stent delivery system offers the advantage of recapture capability. Hot-Axios involves a more intricate manipulation process, whereas Hot-SPAXUS offers a simpler approach akin to conventional metal stent handling, thus streamlining the procedure. An appealing aspect of Hot-SPAXUS is its cost-effectiveness relative to Hot-Axios. The technology related to LAMS continues to evolve, exemplified by the recent introduction and adoption of innovative LAMS designs such as the PLUMBER stent, which incorporates a physician-controlled electrocautery-enhanced delivery system^21^.

Our study has several strengths, including a rigorous analysis of a high-risk population, detailed procedural descriptions, and a focus on both short- and long-term outcomes. However, limitations, such as the retrospective nature of the study, relatively small sample size, potential selection bias, and the absence of a control group for comparison, should be acknowledged. The heterogeneity of the patient population, including various underlying malignancies and comorbidities, introduces potential confounding factors. Additionally, all procedures were performed by EUS intervention expert from a tertiary institution, making generalizing the results to other centers challenging.

In conclusion, our study provides valuable evidence supporting the efficacy and safety of EUS-GBD using Hot-SPAXUS in high-risk surgical patients with AC. The adaptability of Hot-SPAXUS, along with the favorable procedural outcomes and sustained long-term efficacy, makes it a promising tool in the evolving landscape of endoscopic interventions for gallbladder drainage. Further prospective studies with large cohorts and comparative analyses are warranted to validate these findings and establish the broad applicability of the Hot-SPAXUS device in diverse clinical scenarios.

### Supplementary Information

Supplementary Legends.

Supplementary Video 1.

Supplementary Video 2.

## Data Availability

The datasets used and analyzed during the current study are available from the corresponding author on reasonable request.
