# Supplementary material for: Revolutionizing outcomes: endoscopic ultrasound-guided gallbladder drainage using innovative electrocautery enhanced-lumen apposing metal stents for high-risk surgical patients
Source: Sci Rep. 2024 Jun 5;14:12893. doi: 10.1038/s41598-024-63608-5 (PMC11153579; doi:10.1038/s41598-024-63608-5)
Supplement: Supplementary file 1 — Supplementary Legends. [file 41598_2024_63608_MOESM1_ESM.docx]

Video 1. Freehand technique for endoscopic ultrasound-guided gallbladder drainage using Hot- SPAXUS

Video 2. Two-step technique for endoscopic ultrasound-guided gallbladder drainage using Hot- SPAXUS.
